# Gut microbiota dysbiosis in polycystic ovary syndrome: Mechanisms of progression and clinical applications

**DOI:** 10.3389/fcimb.2023.1142041

**Published:** 2023-02-24

**Authors:** Yan Sun, Shouyang Gao, Cong Ye, Weiliang Zhao

**Affiliations:** ^1^ Department of Anesthesiology, China-Japan Union Hospital of Jilin University, Changchun, Jilin, China; ^2^ Department of Obstetrics and Gynecology, China-Japan Union Hospital of Jilin University, Changchun, Jilin, China

**Keywords:** Polycystic ovary syndrome, gut microbiota, insulin, infertility, women

## Abstract

Polycystic ovary syndrome (PCOS) is the most common endocrine diseases in women of childbearing age that leads to menstrual disorders and infertility. The pathogenesis of PCOS is complex and has not yet been fully clarified. Gut microbiota is associated with disorders of lipid, glucose, and steroid hormone metabolish. A large body of studies demonstrated that gut microbiota could regulate the synthesis and secretion of insulin, and affect androgen metabolism and follicle development, providing us a novel idea for unravelling the pathogenesis of PCOS. The relationship between gut microbiota and the pathogenesis of PCOS is particularly important. This study reviewed recent research advances in the roles of gut microbiota in the occurrence and development of PCOS. It is expected to provide a new direction for the treatment of PCOS based on gut microbiota.

## Introduction

PCOS is the most common endocrine diseases with complex etiology and pathogenesis (Glintborg and Andersen, 2010). Among women of childbearing age, the prevalence of PCOS is as high as 5%~10%. It accounts for 50%~70% of anovulatory infertility (Sirmans and Pate, 2013). PCOS is mainly characterized by excessive androgen secretion, ovulation disorders and polycystic ovarian changes, and can be accompanied by abdominal obesity, insulin resistance, impaired glucose metabolism and dyslipidemia (Froment et al., 2022). Its short-term complications include infertility, abortion, preterm delivery and other adverse pregnancy outcomes, which can increase the risk of diabetes, coronary heart disease, endometrial cancer and other diseases in the long term (Hirschberg, 2009). At present, most scholars believe that PCOS is a disease that is controlled by multiple genes and induced by multiple factors. The pathogenesis of PCOS is still unclear, and the clinical treatment effect of many patients is poor.

There are abundant microorganisms in the human intestine. Gut microbiota and the host live together for life and are mutually beneficial. It has become a research hotspot in cancer, immune diseases and metabolic diseases (Hu et al., 2020; Zhao CJ. et al., 2022; Zhao MK. et al., 2022). In recent years, there are also some research reports on the gut microbiota of PCOS patients and its relationship with metabolic abnormalities (Siddiqui et al., 2022). Studies have shown that the gut microbiota of PCOS patients is related to the occurrence and development of insulin resistance, hyperandrogenism, chronic inflammation and metabolic syndrome, and may affect the clinical manifestations of PCOS through short chain fatty acids, lipopolysaccharide, sex hormones and brain-gut axis (Gu et al., 2022; Zhang et al., 2022). In addition, a number of clinical studies have attempted to use fecal microbiota transplantation, probiotics supplementation and traditional Chinese medicine to regulate gut microbiota and explore the possibility of treating certain diseases (Quaranta et al., 2019).

## Changes of gut microbiota composition in PCOS

The composition of human gut microbiota is complex. More and more studies have shown that gut microbiota is closely related to glucose and lipid metabolism (Roessler et al., 2022). Compared with normal people, the composition of gut microbiota in obese, type 2 diabetes and other people changes, and their different flora may participate in inflammatory response, affect the stability of intestinal barrier and improve metabolism (Moser et al., 2022; Sugawara et al., 2022). At present, the pathogenesis of PCOS is still unclear. Many studies have shown that gut microbiota played an important role in PCOS occurrence and development (Duan et al., 2021). A previous study found that decreased intestinal bacteria *Bacteroides*, and increased *Firmicus* and *Proteus* were observed in mice of dehydroepiandrosterone/high-fat diet induced PCOS. Correlation analysis showed that the level of inflammatory factors in mice was correlated with the abundance of gut microbiota (Lin et al., 2021). Compared with the healthy control group, letrozole induced PCOS rats had less intestinal lactobacillus, rumen coccus, and clostridium, and more pullorum (Zhao HY. et al., 2022). Compared with healthy people, the gut microbial diversity of PCOS patients decreased, the composition changed, and the intestinal mucosal barrier was damaged (Lindheim et al., 2017). Compared with non-obese PCOS patients and healthy control population, obese PCOS patients have increased enterobacteria, decreased lactobacillus and bifidobacteria, and changes in gut microbiota are related to inflammation level and insulin resistance (Zhou et al., 2020). Zeng et al. pointed out that there were differences in the composition and structure of gut microbiota in PCOS patients with or without insulin resistance (Zeng et al., 2019). These studies showed that the diversity of gut microbiota and the abundance of related flora in patients with PCOS changed. The gut microbiota may change the stability of intestinal mucosa and then affect its metabolism by participating in the occurrence of inflammatory reaction in patients with PCOS (Zeng et al., 2019).

## Gut microbiota and the pathological mechanism of POCS

Gut microbiota is the “endocrine organ” to maintain human health. Gut microbiota affects the reproductive endocrine system by interacting with estrogen, androgen, insulin, etc (Qi et al., 2021). The typical characteristics of PCOS include abnormal levels of sex hormones, insulin resistance, polycystic changes in the ovary, chronic subclinical inflammation, etc (Szukiewicz et al., 2022). The gut microbiota disorder is involved in endotoxemia, SCFA production, bile acid metabolism, abnormal secretion of brain gut peptides, etc. The above physiological and pathological processes are related to the manifestations of PCOS such as hyperandrogenism, insulin resistance, chronic inflammatory reaction, abnormal levels of brain gut peptide (Li MW. et al., 2022). Therefore, gut microbiota may affect follicular development, sex hormones and metabolic levels through hyperandrogenism, insulin resistance, chronic inflammation, brain-gut axis, etc., and participate in the pathogenesis of PCOS.

## Gut microbiota and insulin resistance

Insulin resistance is one of the most common endocrine characteristics in PCOS patients (Petrillo et al., 2022). 50%~70% of PCOS patients have insulin resistance of different degrees, especially in obese patients. The risk of diabetes in PCOS patients is higher than that in normal women. Insulin resistance and obesity can aggravate the disorder of glucose and lipid metabolism and hyperandrogenin blood in patients with PCOS (Amisi, 2022). Studies have shown that increasing dietary fiber intake and oral butyrate supplements can prevent obesity and improve insulin sensitivity (Mayorga-Ramos et al., 2022). The imbalance of gut microbiota can change the content of SCFAs, especially in PCOS patients with insulin resistance.

Studies have shown that the occurrence of insulin resistance is closely related to gut microbiota disorder (Martinez-Montoro et al., 2022). Zeng et al. compared the gut microbiota of PCOS patients with insulin resistance with that of the healthy control group, and found that the abundance of Prevotella decreased and Bacteroides increased in the former group (Zeng et al., 2019). Gut microbiota may affect bile acid metabolism and lead to insulin resistance. Studies showed that the common Bacteroides in intestinal microorganisms of PCOS patients increased significantly, which may be caused by the reduction of IL-22, insulin resistance and finally PCOS by affecting the level of bile acid synthesis (Qi X. et al., 2019; Qi et al., 2020).

## Gut microbiota and hyperandrogenism

Hyperandrogenism plays an important pathophysiological role in the pathogenesis of PCOS (Witchel et al., 2022). The common clinical manifestations are hirsutism and acne. Studies have shown that hyperandrogenism are related to gut microbiota (Torres et al., 2019). Zhang et al. found that PCOS mice induced by dihydrotestosterone had an increase in the number of chlamydia, but a decrease in the number of Escherichia coli, and affected body mass and fat mass, suggesting that gut microbiota was related to the level of hyperandrogenin in mice (Zhang et al., 2019). Chu et al. found that after transplanting the gut microbiota of male mice to female mice, the basal metabolism of the latter was abnormal, and the testosterone level of mice in the bacterial environment was higher than that of mice in the sterile environment. This indicates that intestinal microbes affect the secretion of testosterone in the body. Markle et al. showed that hyperandrogenism may cause insulin resistance and metabolic abnormalities of PCOS by causing intestinal bacteria enrichment (Markle, 2001). Kelley et al. found the diversity of gut microbiota decreased in Trazole induced Kaohsiung PCOS mouse model (Kelley et al., 2016). Barroso et al. found that the gut microbiota diversity of female rats exposed to a high androgen level environment within 24 hours after birth was reduced, and the risk of metabolic disease in adult offspring was increased, which indicated that the early androgen exposure of female offspring with PCOS might lead to long-term changes in their gut microbiota and metabolic function (Barroso et al., 2020).

## Gut microbiota and chronic inflammation

Leaky gut refers to the dysfunction of the intestinal barrier and has been reported to be related to many diseases. The disorder of gut microbiota could lead to leaky gut. Bacteroides and Escherichia coli in human intestine belong to *Gram-negative* bacteria. LPS is an important component of the cell wall of *Gram-negative* bacteria. Increased LPS absorption from leaky gut has been suggested by several studies. After intestinal mucosal injury, LPS enters the circulation and forms endotoxemia. Through LPS binding protein (LBP), CD14 and bone marrow differentiation factor-2 (MD-2), LPS is recognized and bound by TLR4 (Page et al., 2022). LPS could induce the expression of inflammatory cytokines and inflammatory mediators. The expression of inflammatory factors such as interleukin 6 (IL-6) and interleukin 6 (IL-6) can activate the inflammatory response. Insulin resistance is considered to be the core of metabolic abnormalities in PCOS patients, which promotes the chronic inflammatory state of PCOS patients (Dahan et al., 2022). It has been studied that mice in the two groups were fed with normal and high-fat diets respectively. After 4 weeks, mice fed with high-fat diet became obese and showed signs of insulin resistance. The LPS concentration in blood of mice in the high-fat diet group was 2-3 times higher than that of the control group. LPS was subcutaneously injected into mice in the control group fed with normal diet. After 4 weeks, mice in the control group became obese and produced insulin resistance (Fang et al., 2022).

PCOS patients exhibit chronic inflammatory state (Escobar-Morreale et al., 2011). Macrophages are one of the cells involved in inflammation regulation. A large number of macrophages can be seen infiltrating in ovarian tissue of PCOS patients (Tedesco et al., 2019). In addition, major inflammatory factors in peripheral blood of PCOS patients, such as TNF-α, C-reactive protein, IL-1, IL-6 increased in varying degrees. Other studies have shown that the number of T helper cells in PCOS patients is higher than that in normal women (Yang et al., 2021). Under the stimulation of inflammatory factors such as IL-6, T helper cells 17 can secrete proinflammatory factors, induce the inflammatory state of the body, and lead to adverse outcomes of hyperandrogenism, insulin resistance, and ovulation disorders (Nasri et al., 2018). Wadsworthia in the family Bilophila is a pathogen related to the prophase of inflammatory response, and closely related to the formation of a variety of inflammatory diseases (Burrichter et al., 2021). The gut microbiota of sterile mice transplanted with fecal bacteria from PCOS patients was detected by 16s rDNA sequencing technology. It was found that the level of wadsworthia in mice transplanted with fecal bacteria from PCOS patients was higher than that in mice transplanted with healthy fecal bacteria, suggesting that wadsworthia may participate in the pathogenesis of PCOS through the inflammatory process. Qi et al. found that the abundance of B. vulgatus in the intestine of PCOS patients increased, the level of bile acid, tauroursodeoxycholic acid and glycodeoxycholic acid, metabolites of intestinal bacteria, decreased, and the level of intestinal immune factor IL-22 also decreased (Qi XY. et al., 2019). It is suggested that the mechanism of IL-22 improving PCOS insulin resistance and ovarian function may be related to inhibiting the inflammatory response of ovarian granulosa cells.

## Brain-gut axis and POCS

The brain-gut axis is an information exchange system between the brain and the intestine (Hosie et al., 2022). It is a neuroendocrine immune network formed by the central nervous system, the intestinal nervous system, the hypothalamus pituitary adrenal axis and the intestine. The abnormal metabolism of gut microbiota will lead to abnormal secretion of intestinal endopeptides, cytokines and inflammatory factors (Begum et al., 2022). Various gastrointestinal hormones interact with the brain-gut axis (Yin et al., 2022). Studies showed that the secretion of gastrointestinal hormones in PCOS patients is disordered, and the level of GLP-1 is lower than normal (Bednarz et al., 2022). It can delay gastric emptying, regulate appetite, reduce body mass, and promote pancreatic islets β cell proliferation and stimulation of insulin secretion play an important role in a variety of functions (Cena et al., 2020). Therefore, the brain-gut axis may be a new target for insulin resistance therapy in PCOS patients in the future.

The pathological mechanism of PCOS is not only limited to the dysfunction of the hypothalamus pituitary ovary axis, but also involves the brain-gut axis (Liang et al., 2021). The brain-gut axis is a biphasic signal transduction pathway. The gut and brain are closely connected through the gut brain axis. The brain-gut axis plays an important role in the information exchange system (Li et al., 2023). There is a complex two-way communication system between the central nervous system and the gastrointestinal system (Xu et al., 2022). The microbiota can affect the brain-gut axis in a variety of ways. The gut microbiota can directly stimulate the vagus nerve pathway to send signals to the brain. Various complex information in the intestinal tract can be transmitted to the brain through the synapses formed by the myenteric plexus of the efferent nerve endings and the postganglionic neurons (Wang et al., 2023). At the same time, the gut microbiota forms feedback to the brain by synthesizing hormones and neurotransmitters. For example, the intestinal peptide in the systemic circulation can bind to the homologous receptors of immune cells and the end of the vagus nerve, thus completing the intestinal brain communication (Tan et al., 2022). Gut microbiota disorder may participate in the progress of PCOS through the gut-brain axis (Zhao et al., 2020). Intestinal bacteria can produce SCFA, which is involved in the secretion of brain gut peptides by intestinal endocrine cells, such as glucagon like peptide 1, growth hormone releasing peptide (Ghrelin) and YY peptide. SCFA activates mammalian rapamycin target protein/signal transduction and transcriptional activator signal pathway through G-protein coupled receptor 43 and regulates the expression of brain gut peptide (Zuo et al., 2022). Brain gut peptide (such as Ghrelin) can participate in regulating hypothalamic regulatory nucleus and luteinizing hormone secretion. Ghrelin can also inhibit the excessive synthesis and release of luteinizing hormone by delaying the pulse intensity of pituitary releasing luteinizing hormone, thus participating in regulating the reproductive system function of PCOS (Hoover et al., 2021).

## Treatment of PCOS based on gut microbiome

### Fecal microbiota transplantation

Fecal microbiota transplantation (FMT) is a new treatment for inflammatory bowel disease. FMT is now widely believed to be the transplantation of feces from healthy individuals to the intestinal tract of patients, so as to achieve the purpose of treating diseases by improving and rebuilding the gut microbiota (Hamamah et al., 2022). A previous study demonstrated that the transplantation of fecal flora of healthy mice and Lactobacillus could improve the gut microbiota structure and restore emotional cycle in letrozole induced PCOS rats (Guo et al., 2016). The PCOS mice exposed to healthy intestinal microbes had improved hormone and glucose and lipid metabolism, decreased testosterone, luteinizing hormone, fasting blood glucose and insulin levels, and improved insulin resistance. Regulation of gut microbiota to improve the metabolism of PCOS may be one of the potential options for future treatment of PCOS, but the specific mechanism remains to be explored in depth (Corrie et al., 2021).

### Herbal medicine

In recent years, it has become a new research hotspot to find active ingredients or prescriptions for treating PCOS from herbal medicine, targeting gut microbiota. Meanwhile, Chinese herbal formulas and active ingredients have been used for the treatment of PCOS for a long time (Li J. et al., 2022). And these herbal medicines and their ingredients have the ability to regulate gut microbiota. A previous study demonstrated that Banxia Xiexin Decoction could attenuate PCOS through regulating gut microbiota (Zhao HY. et al., 2022). Furthermore, it has been reported that Guizhi Fuling Wan could inhibit insulin sensitivity in rat model of PCOS by regulating gut microbiota (Zhu et al., 2020; Liu et al., 2021). In addition, berberine has the ability to attenuate PCOS by regulating gut microbiota (Shen et al., 2021). A previous study also demonstrated that quercetin is considered as a potential agent to attenuate PCOS (Vaez et al., 2022). A large number of studies have confirmed that the disorder of gut microbiota may affect the occurrence and development of PCOS (Yu et al., 2022). Therefore, single Chinese medicine and compound medicine targeting gut microbiota provide new targets for the intervention and treatment of metabolic diseases such as obesity, insulin resistance, diabetes, and provide new research directions for the clinical diagnosis and treatment of PCOS.

### Probiotics and prebiotics

In recent years, with the continuous understanding of gut microbiota, the use of microbial agents in the treatment of PCOS has attracted extensive attention (Zhao et al., 2021). More and more evidences show that probiotics, prebiotics, and synbiotics are effective treatment options for PCOS patients (Miao et al., 2021). The study shows that probiotics can restore the gut microbiota diversity of PCOS mice, improve the flora disorder, and improve the reproductive function of mice (Li T. et al., 2022). On the other hand, based on human studies, 60 PCOS patients were randomly divided into two groups and received probiotic supplementation (bifidobacteria, lactic acid bacteria, etc.) and placebo control tests. After 12 weeks, it was found that the sexual hormone binding protein in the test group was increased, the hirsutism score was reduced by R, insulin sensitivity was increased, and the lipoprotein was reduced, indicating that the intervention treatment of probiotics had a certain effect on PCOS patients. In the diet-induced model of diabetes and obese mice, B. lactis B420 strain has been proved to help improve insulin resistance and reduce fat content (Yde et al., 2021). It has been found in clinical studies that the use of B. lactis can improve the sex hormone level of PCOS patients. Probiotics may become an important method to intervene in PCOS obesity in the future. In the traditional correlation studies, the relationship between gut microbiota and disease was studied from a macro perspective. In recent years, in some studies on gut microbiota, by identifying different strains and conducting refined research at the level of specific strains, this kind of research method deserves attention.

### Other drugs

Metformin is one of the commonly used drugs for the treatment of PCOS (Ravn et al., 2022). Diane 35 combined with metformin in the treatment of PCOS is more effective than Diane 35 alone in reducing the level of sex hormones and insulin resistance, improving ovulation, increasing pregnancy rate and the total effective rate of treatment, which suggests that metformin plays an important role in the treatment of PCOS (Zhang et al., 2020). Metformin can reduce the level of inflammation in PCOS patients while improving metabolism (Xue et al., 2019). The effect of metformin on gut microbiota has been gradually recognized in recent years (Wu et al., 2022). The research showed that the composition of gut microbiota of newly treated diabetes patients can be changed. After 4 months of treatment with metformin, the gut microbiota of patients was transplanted into sterile hyperglycemic mice, and the glucose tolerance level of mice was improved, suggesting that metformin can achieve therapeutic effect by changing the gut microbiota (Huang et al., 2022). Metformin intervention can improve the metabolic disorder of obese rats, increase the abundance of Achmania mucophila and Clostridium mucosum, and a total of 18 metabolic pathways (including sphingolipid and fatty acid metabolic pathways) are significantly up-regulated in the gut microbiota. After treatment with metformin, the abundance of Achmania mucophila and gut microbiota producing short chain fatty acids, such as bifidobacteria and Vibrio butyricus, increased in diabetes patients. When metformin is used in healthy people, the diversity of gut microbiota decreases, and some opportunistic pathogens increase, which may be the reason why metformin causes gastrointestinal side effects (Zhang and Hu, 2020). Research showed that after metformin intervention in PCOS mice, intestinal bacteroides and bifidobacteria increase, proteus, helicobacter and parabacilli decrease, and the level of inflammation in the body improves. Correlation analysis shows that changes in gut microbiota are related to inflammatory factors.

Thiazolidinediones are insulin sensitizers, which can be used together when metformin is not effective. The combination of Diane-35 and pioglitazone can significantly improve the hormone level, fasting blood glucose level, insulin level and blood lipid related indicators in patients with PCOS (Cao et al., 2021). Research shows that pioglitazone can reduce the level of inflammation in patients with PCOS, and the combination of pioglitazone and metformin has a more significant effect (Ali et al., 2019). There are few studies on the relationship between thiazolidinediones and the gut microbiota of PCOS patients. Li et al. found that pioglitazone reduced the intestinal microbial diversity of type 2 diabetes mice (Li et al., 2017).

Although the lipid-lowering drugs are not used as the first-line drugs for PCOS, the regulation of blood lipids is still poor after active intervention, and statins should be considered. The study showed that the androgen level in PCOS patients decreased after long-term use of simvastatin, and the blood lipid metabolism improved significantly (Artar et al., 2022). Atorvastatin can improve the hormone level and insulin resistance of obese PCOS patients, and improve their inflammatory level *in vivo* (Chen and Zheng, 2021). However, some scholars pointed out that atorvastatin can reduce insulin sensitivity, and statins should be used cautiously (Sabapathy et al., 2022). Lipid regulating drugs can affect the structure of gut microbiota. The study showed that after atorvastatin intervention in hypercholesterolemic rats, the gut microbiota diversity increased. After taking atorvastatin, the abundance of intestinal proteus bacteria in patients with hyperlipidemia is reduced compared with that in patients without atorvastatin, and the abundance of inflammatory related bacteria is reduced, indicating that statins can improve the gut microbiota composition of patients with hyperlipidemia (Khan et al., 2018).

## Conclusions

In conclusion, there are complex and close interactions between PCOS and gut microbiota. The relationship between gut microbiota and the pathogenesis and pathophysiological process of PCOS needs further study. We hope to clarify the relationship between gut microbiota and PCOS by analyzing the metabolites of gut microbiota in patients with PCOS, which provides a new idea for the prevention and treatment of PCOS and metabolic diseases based on gut microbiota.

## Author contributions

YS, SG and WZ wrote the manuscript. WZ revised the review. All authors contributed to the article and approved the submitted version.
